# A Comparative Evaluation of the Antiproliferative Activity against HepG2 Liver Carcinoma Cells of Plant-Derived Silver Nanoparticles from Basil Extracts with Contrasting Anthocyanin Contents

**DOI:** 10.3390/biom9080320

**Published:** 2019-07-30

**Authors:** Bilal Haider Abbasi, Munazza Nazir, Wali Muhammad, Syed Salman Hashmi, Rashda Abbasi, Lubna Rahman, Christophe Hano

**Affiliations:** 1 Department of Biotechnology, Quaid-i-Azam University, Islamabad 45320, Pakistan; 2 Laboratoire de Biologie des Ligneux et des Grandes Cultures (LBLGC), Plant Lignans Team, INRA USC1328, Université d’Orléans, F 28000 Chartres, France; 3 EA2106 Biomolécules et Biotechnologies Végétales, Université de Tours, 37000 Tours, France; 4 Department of Botany, University of Azad Jammu & Kashmir Muzaffarabad, Azad Kashmir 13230, Pakistan; 5 Institute of Biomedical & Genetic Engineering (IBGE), Sector G-9/1, Islamabad 45320, Pakistan

**Keywords:** anthocyanin, *Ocimum basilicum* L. var. *purpurascens*, cytotoxicity, silver nanoparticles, characterization

## Abstract

Nanotechnology is a well-established and revolutionized field with diverse therapeutic properties. Several methods have been employed using different reducing agents to synthesize silver nanoparticles (AgNPs). Chemical mediated synthetic methods are toxic and resulted in non-desired effects on biological systems. Herein, we, synthesized silver nanoparticles using callus extract of purple basil (BC-AgNPs) and anthocyanin extract deriving from the same plant (i.e., purple basil) (AE-AgNPs), and systematically investigated their antiproliferative potential against HepG2 Liver Carcinoma Cells. The phyto-fabricated AgNPs were characterized by different techniques like UV–visible spectroscopy (UV-Vis), X-ray diffraction (XRD), Fourier transform infrared spectroscopy (FT-IR), Scanning electron microscopy (SEM) and Energy dispersive X-rays (EDX). Morphologically, both types of NPs were found spherical. The average size of BC-AgNPs and AE-AgNPs as revealed through XRD and SEM analyses were calculated as 50.97 ± 0.10 nm and 42.73 ± 1.24 nm, respectively. FT-IR spectral analysis demonstrates the existence of possible phytochemicals required for the capping and reduction of Ag ions. Herein, following solid phase extraction (SPE) coupled to HPLC analysis, we report for the first-time the anthocyanin mediated synthesis of AgNPs and conforming the successful capping of anthocyanin. Small sized AE-AgNPs showed significant cytotoxic effect against human hepatocellular carcinoma (HepG2) cell line as compared to BC-AgNPs. Therefore, the results revealed that the prevalent group of flavonoids present in purple basil is the anthocyanins and AE-AgNPs could be employed as potential anticancer agents in future treatments strategies.

## 1. Introduction

Nanotechnology is an interdisciplinary area of research emerging from innovations in existing domains of science and technology and taking inspiration from the basic and applied sciences of the current era [1,2]. Its interdisciplinary nature enables it to take advantage of core techniques used in various disciplines like chemistry, engineering, physics, and biological sciences and develop novel strategies to manipulate minute particles like atoms and molecules. These manipulations result in the production of nanoparticles (NPs) that may be defined as particles with at least one dimension ranging from 1–100 [3]. Nanotechnology deals with the synthesis and development of a variety of NPs [4]. Many researchers have been captivated to use nanotechnological advancements for the formulation of NPs with greater emphasis on zero valent metallic NPs, because of their multiple benefits in the field of science and technology [5]. Initially, synthesis protocols relied greatly upon the chemical methods to produce NPs [6]; however, issues like towering costs and toxic attributes of synthesized NPs demanded a more feasible alternative to these approaches [7]. Due to the aforementioned issues, the prime focus of researchers turned towards more efficient, eco-friendly and cheaper methodologies that could serve as a replacement for physical and chemical methods in use for NPs synthesis [8]. Nanoparticle biosynthesis by exploiting the green route can prove to be a financial substitute to the physio-chemical approaches [9]. Utilization of green route also has an added advantage of non-toxicity, and, therefore, a viable option for their prospective use in several biomedical applications [10]. Among biological methods, plant-based biosynthesis of NPs is considered a gold technique, due to easy availability and diverse nature of plants [11]. The potential of plant extracts to produce NPs that have definite size and shape, as well as composition, is of great importance. Phytochemicals that are readily available in plant extracts are utilized in the green approach as the natural stabilizing and reducing agents for the biosynthesis of NPs [12]. 

*Ocimum basilicum* L. var. *purpurascens* (Purple basil) is an aromatic medicinal plant from the *Lamiaceae* family. This plant species has great economic value and is equally famous in traditional medicine, due to its widespread pharmaceutical potential [13]. Basil is the main source of valuable compounds, such as sesquiterpenes, phenolic acids, anthocyanins, phenylpropanoids and monoterpenes [14]. One of the major sources of anthocyanins is purple pigmented basil [15]. Anthocyanins are considered as plant best-characterized secondary metabolites, and are a dominant class of flavonoids [16,17,18]. Anthocyanins perform diverse functions within a plant body, such as pathogenic defense, UV protection, and DNA protection. Moreover, anthocyanins are also well known for antioxidant, anticancer and anti-inflammatory properties [19]. 

There are several studies available on plants, and their extracts mediated AgNPs formation, however, to the best of our knowledge, the role of anthocyanins as reducing and capping agents for AgNPs synthesis have not been explored previously. Herein, we report the successful synthesis of AgNPs via callus extract of purple basil (BC-AgNPs), and purple basil derived anthocyanin extract (AE-AgNPs). The configuration and dimensions were examined by SEM, while XRD and FT-IR confirmed the chemical nature and phase of the synthesized NPs. Solid phase extraction procedure (SPE) followed by HPLC successfully verified the capping of anthocyanin. As proof of the concept, these nanoparticles were evaluated for their anticancer potential.

## 2. Material and Methods

### 2.1. Chemicals

All the chemicals used in the present study were of analytical grade quality and purchased from Thermo (Illkirch, France). The deionized water was produced using a milli-Q water purification system (Merck Millipore, Molsheim, France). Prior to their use for analysis, all solutions were filtered through 0.45 µm nylon syringe membranes (Merck Millipore, Molsheim, France). All commercial standards were purchased from Sigma-Aldrich (Saint-Quentin Fallavier, France).

### 2.2. Biological Synthesis of AgNPs

To prepare callus extract, 15 g of freshly obtained callus produced as described in [20] was introduced into Erlenmeyer flask containing 150 mL distilled water. Aluminum foil was used to cover the flask and then kept in an incubator for a week at 45 °C. Filtration of the extract was done via Whatman filter paper and stored at 4 °C in the refrigerator until further use.

**Figure 1:**
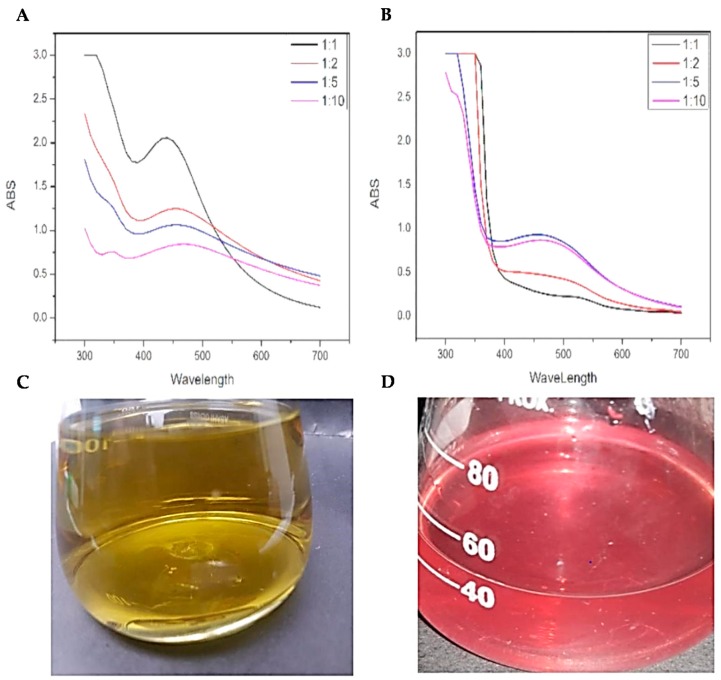
(**A**) UV-Vis spectral analysis of BC-AgNPs; (**B**) UV-Vis spectral analysis of AE-AgNPs; (**C**) optical observation of green synthesized callus extract; (**D**) optical observation green synthesized anthocyanin extract.

Anthocyanin is a polar material, thus soluble in a variety of solvents [21]. To prepare anthocyanin extract (AE) from callus, the callus was macerated properly and introduced into methanol. Upon addition of 1% HCl, the mixture was kept at ambient temperature till the advent of a light purple color which serves as an indication of extraction of anthocyanins (Figure 1D). Anthocyanin’s presence was confirmed by observing absorption peak UV-visible spectrophotometer (Varian, Le Plessis-Robinson, France) at 530 nm [22]. Whatman filter paper was used for filtration of the resulting mixture and kept the mixture at 4 °C in the refrigerator until further use.

To synthesize AgNPs, protocols of [10] was used. Two types of NPs were synthesized from callus of purple basil. Briefly, AgNO_3_ (1 mM) was added to BC (callus), and AE (anthocyanin) extracts in different ratios (1:1, 1:2, 1:5 and 1:10) where the amount of BC and AE was kept constant. The mixture was then stored at 25 °C for 24 h. UV-vis spectrophotometer was used after 24 h to confirm the biosynthesis and absorbance of BC and AE mediated AgNPs.

### 2.3. Structural and Optical Characterization of Green Synthesized AgNPs

Characterization is very important to confirm the synthesis of nanoparticles and have a better understanding of their morphology and nature. For this purpose, the synthesized AgNPs were subjected to some of the common characterization techniques.

The initial confirmation of AgNPs synthesis was done by UV-Vis spectrophotometric (HALO DB-20) analysis of precursor salt and plant extracts. The crystallinity of the biosynthesized dry powder AgNPs was characterized through XRD analysis (Xpert Pro, Malvern Panalytical, Eindhoven, Netherlands) in the range of 20–80° through Cu-Kα radiation having a wavelength of 1.5406 Å. To calculate size Debye–Scherrer equation was used.

D = kλ/βθcos

Whereas k is equal to 0.94, λ is 1.5418 Å, β is the full width at half maximum radians, and the Bragg’s angle is denoted by θ.

ATR-FTIR (Model TENSOR II, Bruker, Ettlingen, Germany) analysis of the AgNPs powder was performed for the identification of functional groups.

Surface morphology and elemental composition were visualized through SEM (SIGMA model, MIRA3, TESCAN, Fuveau, France) and EDX detector (TESCAN, Fuveau, France).

### 2.4. Solid Phase Extraction Procedure

For the separation and quantification of compounds from NPs, solid phase cartridges (Sep Pak Plus C18 cartridges) were used (Table 1). Visiprep SPE vacuum manifold (12-port model) from Supelco (Supelco, Saint-Quentin Fallavier, France) was used in the extraction procedure. Extraction was done according to a previously established method [23].

### 2.5. HPLC Analysis

Samples were loaded into HPLC vials and quantified using Varian high-performance liquid chromatography system equipped with Varian Prostar 230 pump, Meta chem Degasit degasser, Varian Prostar 410 autosampler and Varian Prostar 335 Photodiode Array Detector (PAD) and driven by Galaxie version 1.9.3.2 software (Varian, Le Plessis-Robinson, France). The HPLC grade standards for isolated compounds, i.e., caffeic acid, chicoric acid, rosmarinic acid, cyanidin, and peonidin, were acquired from Sigma Aldrich. The technique used for separation is adapted from [24]. For separation, Hypersil PEP 300 C18, 250 × 4.6 mm, 5 µm particle size prepared by a guard column Alltech, 10 × 4.1 mm was used at 35 °C. Compounds detection was achieved at wavelengths of 320 nm and 520 nm. The mobile phase was composed of A = HCOOH/H_2_O, pH = 2.1, B = CH_3_OH (HPLC grade solvents). Mobile phase composition varied throughout 1-h per run, with a nonlinear gradient 8% B (0 min), 12% B (11 min), 30% B (17 min), 33% B (28 min), 100% B (30–35 min) and 8% B (36 min) at a flow rate of 1 mL/min. Among individual runs, a 10-min re-equilibration time was used. Quantification was done to rely on an assessment of retention times and reliable reference standards. All the samples search was completed thrice. The results were stated as µg/mg DW of the sample.

### 2.6. In Vitro Cytotoxicity of AgNPs against HepG2 Cell Line

The protocol of [12] was used for evaluating the anticancer potency of biosynthesized AgNPs. Briefly, culturing of HepG2 (Human hepatocellular carcinoma cells) (ATCC HB-8065) was carried out in Dulbecco’s modified eagle medium (DMEM) containing 10% Fetal Calf Serum (FCS), 1 mM Na-pyruvate, 2 mM L-glutamine, 100 μg/mL streptomycin and 100 U/mL penicillin. Cells were placed in humidified (5% CO_2_) atmosphere, and the temperature was adjusted to 37 °C. 0.5 mM trypsin/EDTA was used for cell harvesting. Sulforhodamine B (SRB) assay was used to test the potency of BC-AgNPs and AE-AgNPs against HepG2 cultures. For this purpose; colloidal solution of AgNPs in deionized water was used. Twelve thousand cells of HepG2 per well were cultured into 96 well plates and allowed to adhere at 37 °C for a day. Cells (80–90% viable) were then exposed to BC-AgNPs and AE-AgNPs (200 µg/mL) separately for a day at 37 °C. The cells were then incubated for an hour at 4 °C after the addition of 50% pre-chilled Trichloroacetic acid (TCA) followed by rinsing three times with deionized water to finish cell fixation. Cell staining was performed with SRB dye (0.01%) at room temperature. After 30 min, the plates were washed with acetic acid (1%) to remove the extra dye. Non-treated cells (NTC) served as a control for the experiment. Photographs were captured by an Olympus CK2 light microscope with a digital camera. SRB dye was later dissolved in 100 µL/well of 10 mM Tris (pH 8) for 5 min at ordinary temperature. The absorbance of the plates was recorded at 565 nm with the help of Microplate reader (Platos R, 496. AMP, AMEDA Labordiagnostik GmbH, Graz, Austria). The following formula was then used to calculate %viability relative to untreated control:

Cell viability (%) = (Absorbance of test sample − Absorbance of control)/(Absorbance of untreated cells − Absorbance of media) × 100.

While, % inhibition was calculated with the formula:

% Inhibition = 100 − % Cell Viability.

### 2.7. Statistical Analysis

All experiments were performed thrice. For statistical analysis and making graphs, Origin software (v8.5) was used. Analytical data were presented as means ± SD using Microsoft Excel Program.

## 3. Results and Discussion

### 3.1. Characterization of Biosynthesized AgNPs

#### 3.1.1. UV-Vis Spectrophotometric Analysis 

To confirm the biosynthesis of AgNPs, and to find the most suitable ratio for the biosynthesis of AgNPs using BC and AE, the mixture of AgNO_3_ and the extracts mixed in different ratios were characterized with the aid of UV-Visible spectrophotometer. Maximum absorbance was observed for 440 nm wavelength for BC-AgNO_3_ at 1:1 (Figure 1A) and for AE-AgNO_3_ (Figure 1B) at 1:5. These ratios were selected for preparation of AgNPs. Figure 1 shows the spectrophotometric analysis of callus and anthocyanin extract mediated AgNPs at different ratios. 

#### 3.1.2. XRD Analysis

**Figure 2:**
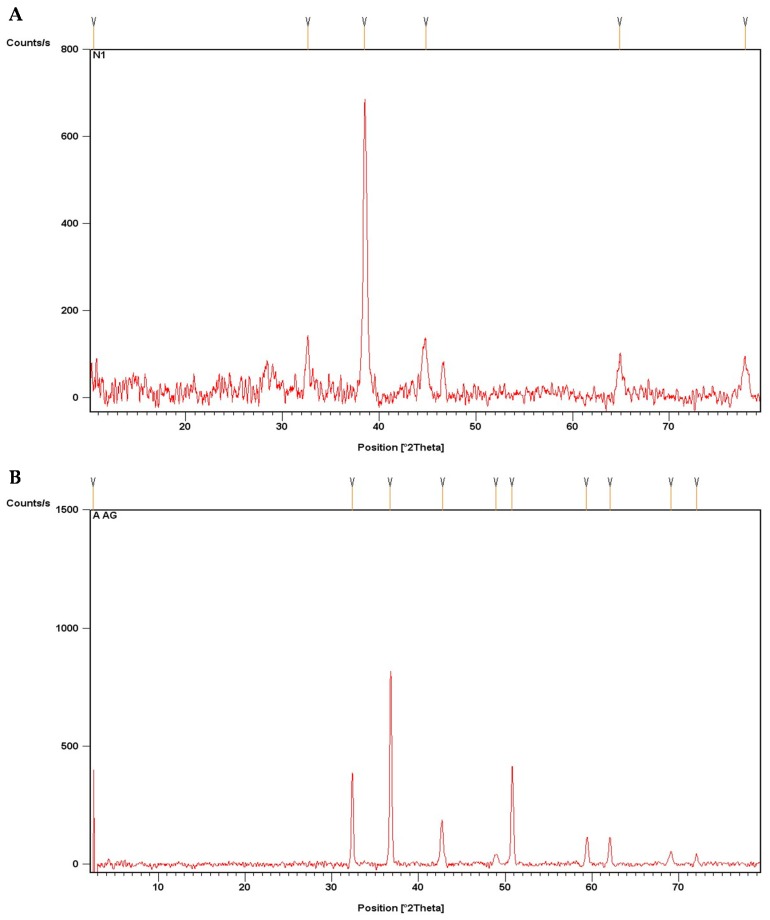
XRD analysis of AgNPs: (**A**) XRD image of BC-AgNPs; (**B**) XRD image of AE-AgNPs.

For phase identification and crystalline nature of biosynthesized AgNPs, powdered BC-AgNPs and AE-AgNPs were subjected to XRD analysis. AgNPs showed peaks at 37.976°, 44.1069°, 64.1987°, and 77.2926°, while AE-AgNPs showed peaks at 32.3635°, 36.7353°, 42.7714°, 48.9495°, 50.7722°, 59.3511°, 62.0693°, 69.1236°, and 72.0772°. The peaks of BC-AgNPs corresponds to (111), (200), (220) and (311) planes of AgNPs Bragg’s reflection and prove that the synthesized AgNPs had a face-centered cubic crystalline structure. Similarly, the peaks of AE-AgNPs at 32.3635°, 36.7353°, 48.9495°, 59.3511°, 69.1236°, and 72.0772° closely corresponds to (122), (111), (231), (241), (220) and (311) planes of AgNPs Bragg’s angles. Apart from these peaks, a few unknown peaks were also obtained, which might be due to certain impurities within the synthesized nanoparticles [25] also obtained similar sort of XRD data for AgNPs. Size calculated by Scherrer equation of AE-AgNPs 50.9 nm and BC-AgNPs 43.6 nm. Figure 2A,B show the graph from the XRD analysis of the AgNPs.

#### 3.1.3. FTIR Analysis

**Figure 3:**
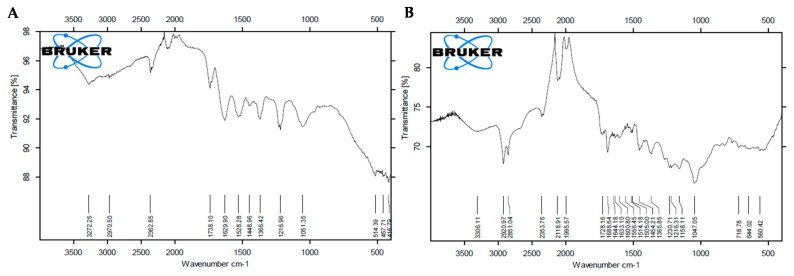
(**A**) FT-IR image of BC-AgNPs (**B**) FT-IR image of AE-AgNPs.

A plethora of plants’ secondary metabolites have been reported to be involved in the capping of AgNPs. To confirm the involvement of secondary metabolites in the capping of AgNPs synthesized in the current study, the powdered AgNPs were subjected to FTIR analysis. FTIR analysis of BC-AgNPs and AE-AgNPs showed distinct peaks at several wavelengths ranging from 3500–500 cm^−1^ (Figure 3A,B). Peaks at 3272.2 cm^−1^, 2970.5 cm^−1^, 2362.8 cm^−1^, 1738.1 cm^−1^, 1629.9 cm^−1^, 1528.2 cm^−1^, 1448.9 cm^−1^, 1366.4 cm^−1^, 1216.9 cm^−1^, 1051.3 cm^−1^, 514.3 cm^−1^, 457.7 cm^−1^ and 416.7 cm^−1^ were obtained for BC-AgNPs. AE-AgNPs showed peaks at 3306.1 cm^−1^, 2920.9 cm^−1^, 2851.0 cm^−1^, 2353.7 cm^−1^, 2118.9 cm^−1^, 1995.5 cm^−1^, 1728.1 cm^−1^, 1688.6 cm^−1^, 1644.1 cm^−1^, 1633.1 cm^−1^, 1600.8 cm^−1^, 1556.4 cm^−1^, 1514.1 cm^−1^, 1505.0 cm^−1^, 1454.2 cm^−1^, 1365.8 cm^−1^, 1230.7 cm^−1^, 1216.8 cm^−1^, 1158.1 cm^−1^, 1047.0 cm^−1^, 718.7 cm^−1^, 644.0 cm^−1^ and 560.4 cm^−1^. ATR-FTIR spectra of Purple basil BC-AgNPs and AE-AgNPs showed strong C–N aliphatic ammines in the range of 1051.5–1216.9 and 1020–1250 cm^−1^ respectively. Alkynes (-C≡C-) were also identified in both types of nanoparticles at 3272.2 (BC-AgNPs) and 2118.9 cm^−1^ (AE-AgNPs) wavelengths [26,27]. Similarly, in both samples, prominent bands were observed at around 1448.9, 1454.2, 1738.1, 1688.6 cm^−1^. The observed peaks denote C-H alkanes group and C=O aldehydes linkages, respectively. However, alkyl halide C-Br, (560.4–644.0 cm^−1^), C-Cl (718.7 cm^−1^) and α-*β* ester (1728.1 cm^−1^) stretches were obtained for the only anthocyanin mediated silver nanoparticles. These bands denote stretching vibrational bands that can be ascribed to the presence of polyphenolic compounds, such as flavonoids or hydroxycinnamic acids derivatives [28,29,30].

#### 3.1.4. SEM and EDX Analysis

**Figure 4:**
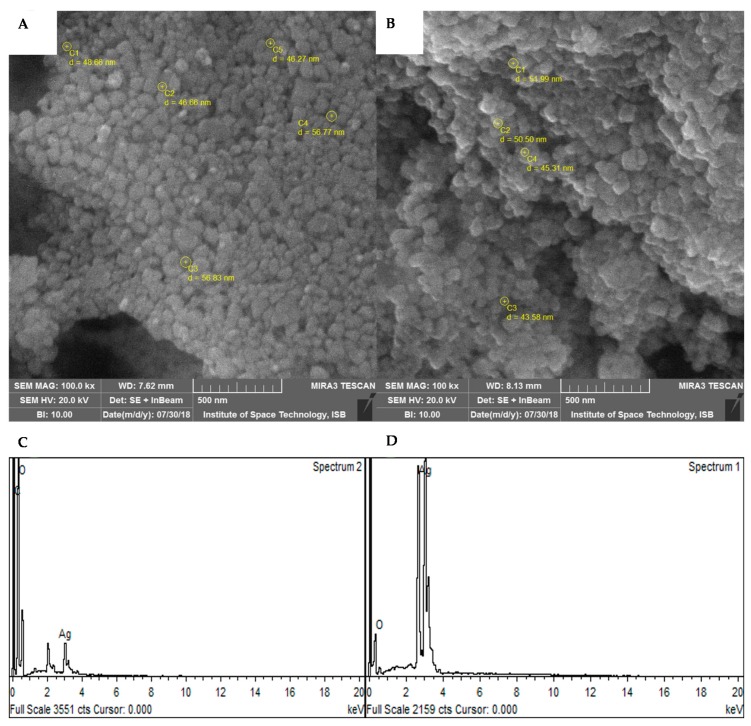
SEM and EDX analysis of AgNPs where (**A**) SEM image of BC-AgNPs (**B**) SEM image of AE-AgNPs (**C**) EDX image of BC-AgNPs (**D**) EDX image of AE-AgNPs.

SEM and EDX are powerful tools for the study of structural morphology and purity of nanoparticles. SEM and EDX analysis of the synthesized AgNPs revealed that both BC-AgNPs and AE-AgNPs had spherical morphology (Figure 4A,B). Both samples AgNPs were in the highly aggregated state. The average size of BC-AgNPs and AE-AgNPs as revealed through SEM analysis was calculated to be 51.04 and 41.85 nm, respectively, which is in a close proximity with XRD analysis. EDX analysis also confirmed the presence of AgNPs with strong peaks of silver. No peaks of any other metal were found in EDX analysis, which refers to the high purity of the synthesized AgNPs.

### 3.2. SPE and HPLC

For isolation, separation and/or adsorption of one or more components from a liquid phase (sample) onto a stationary phase (resin or sorbent), solid phase extraction (SPE) is used. It is a form of digital chromatography. SPE is considered as the most dynamic method for selective and rapid sample preparation prior to analytical chromatography for the past 20 years. SPE has become one of the most extensively used technique for numerous samples [31,32]. This technology has many advantages, such as easy operation, high enhancement factor, fast separation phase, effective matrix interference, maximum recovery, minimum solvents consumption and cost-effective [33,34].

In recent years, nanoscale metal oxides have gained considerable attention from the researchers, as compared with conventional adsorbents, given their high surface area and rapid absorption kinetics.

More than a few reports confirmed that the NPs were high adsorbent materials and used in SPE procedure for extraction [35]. Likewise, in order to extract pesticides from water SPE cartridge based on chitosan-metal oxide nanoparticles (Ch-MO NPs) was developed [36]. Whereas, in the current study, silver nanoparticles (AgNPs), including callus extract nanoparticles (BC-NPs) and anthocyanin mediated nanoparticles (AE-AgNPs) were synthesized from purple basil. The SPE cartridges were used in the extraction of hydroxycinnamic acid derivatives and anthocyanin from nanoparticle samples and were determined by HPLC system. This procedure includes the quantification of these compounds from BC-Ag NPs and AE -NPs and compared with the commercial (CM) Ag-NPs. (Table 1). We observed that BC-Ag NPs showed anthocyanins (cyanidin and peonidin), caffeic acid, rosmarinic acid and chicoric acid in a specific amount, which indicates that capping might be due to these secondary metabolites. As phenols and flavonoids act as stabilizing and reducing agents for NPs synthesis, these components are highly responsible for reducing silver ions to Ag NPs [37]. However, in the case of AE-NPs, interesting results were observed. HPLC analysis indicated the presence of only anthocyanin (cyanidin and peonidin), while no other compounds were detected as compared to BC-AgNPs. This is the first and novel report on anthocyanin mediated silver NPs synthesis. These results indicated that the active reducing agent in AE-AgNPs was anthocyanins, which results in the reduction of metal ions into metal NPs. In the literature, many reports are available which address anthocyanin as reducing and stabilizing agent in callus extract [38]. Green synthesis of AgNPs preparation using berries extracts rich in anthocyanins has been reported in the literature [38]. Anthocyanins are well known natural UV protectants, pathogens inhibitors, and food coloring compounds from plants [39]. This study also verified that anthocyanin serve as reducing and stabilizing agents. Here, we report on a novel plant in vitro system for the preparation of an anthocyanin-rich extract from purple basil used for the green synthesis of NPs. For this purpose, a method based on SPE was used for the anthocyanin-rich extract, and HPLC analysis confirmed the effective capping. This protocol provided several benefits over other techniques as fast, simple, eco-friendly nature and cost-effective.

### 3.3. Anticancer Potential Evaluation

**Figure 5:**
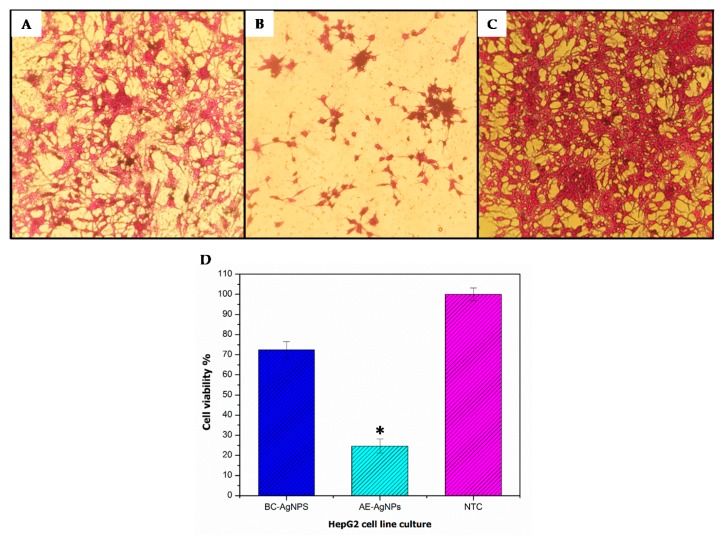
Cytotoxic effects of biologically synthesized AgNPs on HepG2 cells upon 24-h treatment with 200 µg/mL concentration. Untreated cells were included as controls. Microscopic images of HepG2 cells (treated and untreated). Magnification = 200×. (**A**) Image of BC-AgNPs cytotoxic activity; (**B**) image of AE-AgNPs cytotoxic activity; (**C)** image of Non-treated HepG2 cells; (**D**) percentage viabilities of cells relative to untreated control (Mean ± SD). Each sample was studied in triplicates (biological replicates), and the experiment was performed twice (technical replicates). * *p* ≤ 0.05 (two tailed t-test) when compared to NTC (non-treated cells).

Around 70% of liver cancer cases are diagnosed as hepatocellular carcinoma (HCC) worldwide. Since most of the cancers, including HCC are asymptomatic, diagnosis is made in later stages where treatment strategies are rendered useless, resulting in higher mortality rates in patients [40]. Therefore, novel strategies for the treatment of such cancers are expected. In our study, we tested BC-AgNPs and AE-AgNPs against HepG2 cell lines through the SRB assay (Figure 5). AE-AgNPs showed significantly (*p* value ≤ 0.05) higher cytotoxicity (24.70 ± 4.5%) when compared to untreated control (Figure 5D). On the contrary, cells treated with BC-AgNPs showed non-significant results with the viability of 72.49 ± 5.8%, recorded at 200 µg/mL dose. Similarly [41], studies pointed out the cytotoxicity of AgNPs, in a cancer cell line with 300 µg/mL of an LD_50_ value. AgNPs have been reported to induce ROS generation, thus damaging cellular components, followed by cell death [42]. In current study spherical shaped anthocyanin mediated AgNPs of purple basil showed noticeable cytotoxicity against HepG2 cell line, in good agreement with the results observed with *Piper nigrum* extract mediated biosynthesized AgNPs in vitro cytotoxicity against HepG2 cells [43]. In the same way, the current results were in harmony with the results reported for *O. vulgare* extract mediated AgNPs cytotoxicity on HeLa cell line [44].

Nonetheless, we report for the first time the cytotoxicity of green synthesized AgNPs using purple basil callus extract and anthocyanin extract against HepG2 cell lines. The improved cytotoxicity of purple basil may be due to the manifestation of several bioactive compounds, such as rosmarinic acid, caffeic acid, chicoric acid cyanidin or peonidin as capping agents in green synthesis of AgNPs. The present study indicated that AE-AgNPs exhibited high anticancer activity then BC-AgNPs suggesting that anthocyanin alone as capping agent have more potential of cellular toxicity against HepG2 cells. The report is the first to depict the cytotoxic effect of anthocyanin mediated AgNPs against HepG2 cell line. Further studies are now required to recognize the possible mechanism, underlying the anticancer activity and potential therapeutic use of these nanoparticles. In particular, to evaluate the selectivity of these NPs towards cancer cells.

## 4. Conclusions

Our results concluded that *Ocimum basilicum* L. var. *purpurascens* (purple basil) possesses essential bioactive compound responsible for its therapeutic potential. Anthocyanins are the main flavonoid products of purple basil having antioxidant properties, due to which it has been extensively used in medicines and serves as an antibacterial, antifungal and anticancer agent. The characterization with FT-IR, UV-Vis spectroscopy, SEM, XRD and EDX confirmed nanoparticles formation, whereas SPE/HPLC verified the successful capping by secondary metabolites. The synthesized AgNPs displayed promising anticancer action. To this end, the in vitro cytotoxicity of AE-AgNPs against HepG2 cell line was significant, with 75% mortality at 200 µg/mL. These results suggested that anthocyanin mediated nanoparticles can be employed as a fast, simple, cost-effective, eco-friendly efficient agent to treat fatal diseases like cancer. However, further research is now required to exploit the underlying mechanism.

## Figures and Tables

**Table 1:** Detection of phenylpropanoids metabolites by HPLC from BC-AgNPs, AE- AgNPs and CM-AgNPs. nd: Not detected.

Phenylpropanoid Metabolites (µg/g)	BC-AgNPs	AE-AgNPs	CM-AgNPs
Caffeic acid	3.31 ± 0.01	nd	nd
Chicoric acid	68.64 ± 2.05	nd	nd
Rosmarinic acid	235.35 ± 4.55	nd	nd
Cyanidin	0.47 ± 0.006	1.56 ± 0.074	nd
Caffeic acid	3.31 ± 0.01	0.85 ± 0.0019	nd

## Abbreviations

CE: Callus extract, AgNPs: Silver Nanoparticles, AE: Anthocyanin extract BC-AgNPs: Basil callus derived silver nanoparticles, AE-AgNPs: Basil anthocyanin derived silver nanoparticles, UV-vis: Ultraviolet-visible, FTIR: Fourier-transform infrared, XRD: X-ray diffraction, SEM: Scanning electron microscopy, EDX: Energy dispersive X-rays, HepG2: Human hepatocellular carcinoma cell line, DMEM: Dulbecco’s Modified Eagle’s medium, SRB: Sulforhodamine B, TCA: Trichloroacetic acid.
